# Methods to measure biological sounds and assess their drivers in a tropical forest

**DOI:** 10.1016/j.mex.2022.101619

**Published:** 2022-01-14

**Authors:** Johan Diepstraten, Jacques Keumo Kuenbou, Jacob Willie

**Affiliations:** a Animal Behaviour and Cognition, Department of Biology, Faculty of Science, Utrecht University, The Netherlands; b Association de la protection des grands singes, Cameroon; c Centre for Research and Conservation, Royal Zoological Society of Antwerp, Belgium

**Keywords:** Ecoacoustics, Audio recordings, Human listeners, Audiomoth, Transects, Mammal surveys, Bird surveys, Human disturbance

## Abstract

The study of soundscapes and biological sounds is becoming the focus of increasing scientific attention. Studying biological sounds involves the deployment of acoustic sensors to record sounds and the identification of animal species and other sources of sound in audio recordings. In addition, data extracted from audio recordings may be pooled together with ecological and human activity data to investigate the drivers of biological sounds. We provide a detailed method description of our study on biological sounds in a tropical forest and their drivers along a gradient of disturbance in Southeast Cameroon. We designed and implemented a research protocol to:

• make large scale audio recordings and identify animal species detected;
• collect ground-truth data on mammal and bird species;
• collect climate, habitat, and human activity data and describe remoteness and accessibility.

## Graphical abstract

**Figure:**
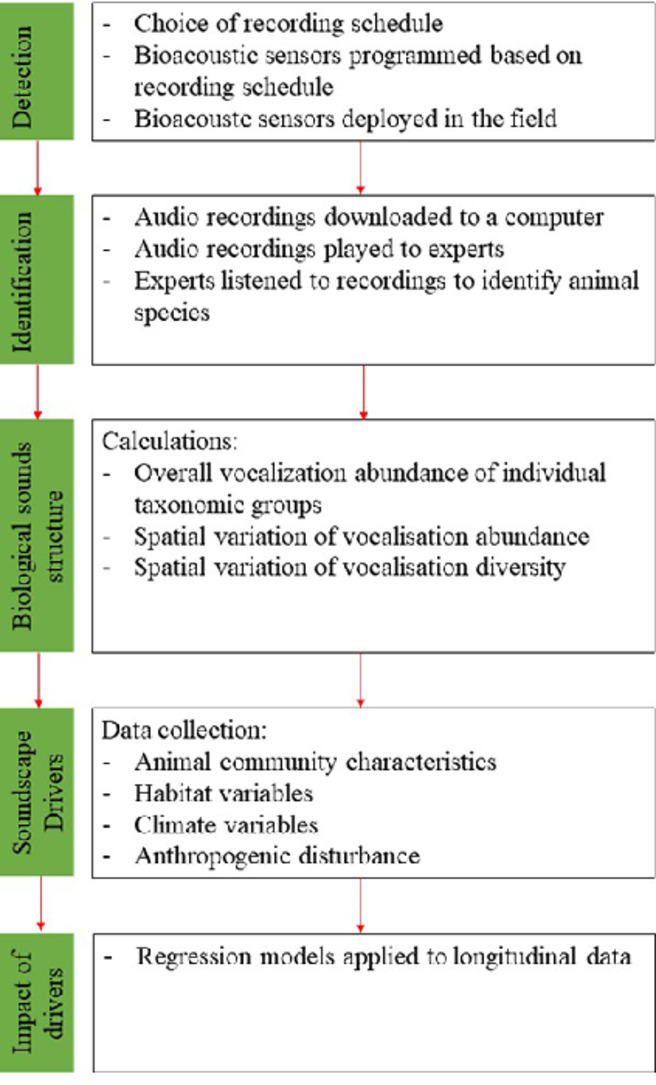

Specifications table

**Table:** 

Subject Area:	Environmental Science
More specific subject area:	Ecoacoustics
Method name:	Passive acoustic monitoring and analysis
Name and reference of original method:	N.A.
Resource availability:	AudioMoth: https://www.openacousticdevices.info/ iNext: https://chao.shinyapps.io/iNEXTOnline/

## Methods

### Study area

**Fig. 1:**
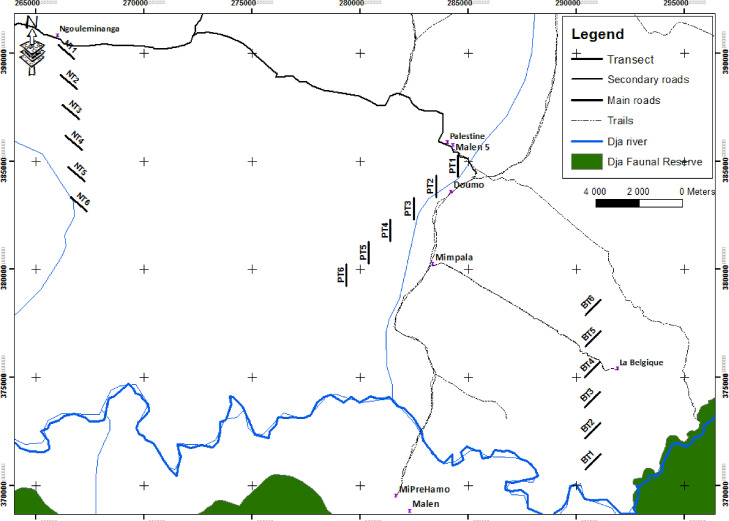
Location of the three study sites and the adjacent villages in the northern periphery of the Dja Faunal Reserve. Six 1-km transects were opened in each site using a cascading design. The transects were cut with a constant compass bearing of 140°, 180°, and 45° in Ngouleminanga, La Palestine, and La Belgique, respectively.

This study was conducted in the northern part of the Dja Faunal Reserve's buffer zone in Cameroon. Data were obtained in three study sites (Ngouleminanga, La Palestine, and La Belgique) that differ in land-use type and conservation management (Table 1; Fig. 1). Since the overall level of disturbance in a site depends on these two factors, these three sites are expected to represent a gradient of disturbance. Logging was absent throughout the study area.

**Table 1:** Overview of how the three study sites represent a disturbance gradient.

Site	Population size (#)	Land-use type	Conservation management	Disturbance
Ngouleminanga	130	Community forest	Absent	High
La Palestine	176	Community forest	Present	Medium
La Belgique	182	Forest management unit	Present	Low

Ngouleminanga is a forest site and eponymous village located 24 km north of the Dja Faunal Reserve. The village is easily accessible by motorised vehicles and supports an estimated population of 130 inhabitants [8]. Ngouleminanga is used as a community forest (CF), indicating that local communities can derive their livelihoods in a sustainable manner from this forest [4]. Furthermore, no active conservation management is present in Ngouleminanga. La Palestine is a forest site adjacent to the villages Malen V, Doumo-Pierre, and Mimpala. Together, these villages have an estimated population size of 350 inhabitants [8]. Malen V is easily accessible by motorised vehicles, as opposed to Doumo-Pierre and Mimpala. La Palestine is located 17 km north of the Dja Faunal Reserve. The entire site is considered to be a CF. However, active conservation management in this area has been conducted by APGS since 2001. APGS focusses on the protection of wildlife through conservation-applied research and support to the local community. The organisation promotes educational programmes within the local community to increase their awareness of the local natural environment. La Belgique is a research site founded and managed by APGS and located 2 km north of the Dja Faunal Reserve. The site is only accessible by foot and the nearest villages (Doumo-Pierre and Mimpala) house approximately 180 inhabitants [8]. The forest is officially unprotected and is classified as a forest management unit (FMU), meaning that it is property of the state and can be used for timber production purposes [1]. However, APGS has signed an agreement with local villages prohibiting human activities, such as hunting, within the research site. Accordingly, the presence of APGS has proven to counteract the negative effects of hunting and deforestation in the area [18], [20].

### Data collection

Field work was conducted between February and May 2020. During this time, acoustic measurements were performed for the detection and identification of individual vocalising species and the determination of biological sounds. Eighteen AudioMoth bioacoustics sensors were used throughout the study area (6 per site). Six transects of 1 km each were opened in each site and one sensor was deployed in the middle of every transect, at the 500-m mark [13]. To create enough space between the transects within each site, a cascading design was used. In this design, the distance between the start and the end of the adjacent transects was also 1 km. This resulted in a √2-km distance between sensors. The transects were cut with a randomly chosen, constant compass bearing of 140°, 180°, and 45° in Ngouleminanga, La Palestine, and La Belgique, respectively (Fig. 1).

**Fig. 2:**
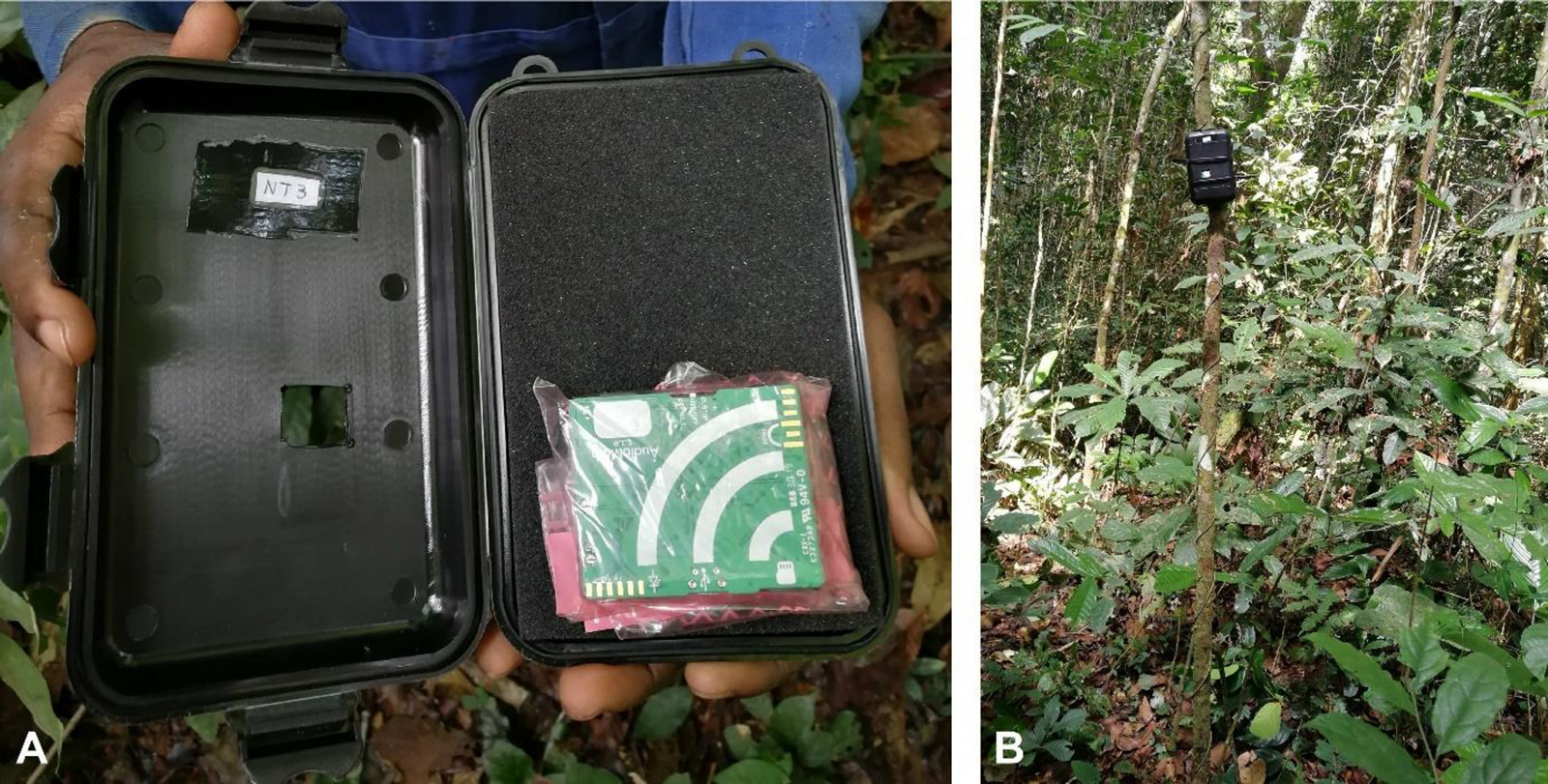
Audio sampling design. **A)** An AudioMoth bioacoustics sensor wrapped in a zip lock bag, placed in a protective, labelled case with foam in the back to keep the sensor in place and a small hole in the front at the location of the microphone. **B)** A sensor deployed in the forest, attached to a tree at a height of 2 m, orientated at 90°.

Different setups were tested to protect the sensors against rain and animals. The setup where all sensors were kept in zip lock bags within a protective case resulted in the clearest audio quality while also protecting the case. Furthermore, at the location of the sensors’ microphone, a small hole was made in the case to ensure optimal audio quality of the recordings (Fig. 2A). For consistency, all sensors were attached to a small tree at a height of 2 m, oriented at 90° (Fig. 2B).

Bats, which produce ultrasonic sounds, were not included in this study. Therefore, only sounds within a human audible range were recorded. For proper recording, the sampling rate of the sensors should be over twice as high as the highest frequency of interest, or Nyquist frequency [3]. Therefore, the sampling rate of all sensors was set to record at 48 kHz. Since the tested environment did not show high levels of noise, recordings were made at 30.6 dB [13]. All sensors were set to record the first minute of every hour, resulting in 24 min of sound recordings per transect per day.

In total, 20 485 min of sound were obtained from the three sites. 5949, 7712, and 6824 min were recorded in Ngouleminanga, La Palestine, and La Belgique, respectively (Table 2). All recordings made during rainy periods were excluded from the listening process and the analyses. This was the case for 1895 audio files. To expedite the listening process, all recordings made during the night were screened by JD beforehand. Only night recordings that contained vocalisations other than those of insects, amphibians or western tree hyraxes (*Dendrohyrax dorsalis*), which were all easily recognisable after some training, were played to the local expert listeners for identification. All remaining recordings were played to two local villagers who could identify the audible species. These listeners consistently identified the same sounds as being the same species. The listening process took two months. On average, 340 audio recordings were listened to each day. For each recording, these local experts were asked to independently write down the names of all the species they heard in their local language, Badjué. The English and scientific translation of many of the local names were already known, if not, the local experts were asked to pinpoint the species in local identification guides [2], [14]. When the local experts did not unanimously agree on the identification of an audible species, they were asked to reach a consensus through discussion or replaying a recording as many times as necessary. To avoid bias, they were kept uninformed about the site in which each recording was made. Since this study used mammals and birds to evaluate the impact of ecological and anthropogenic factors on vocalisation patterns, vocalisations from these taxa were identified by species. Vocalisations from amphibians and insects were identified by class. Unidentifiable animal sounds were noted down as ‘Animal unknown’ or, if the local experts were sure that the sound was produced by a bird, ‘Bird unknown’.

**Table 2:** Recording schedule and number of recordings per sensor.

Site	Sensor	# Recordings	Start date	End date
La Belgique	BT1	1244	26-2-2020	26-4-2020
La Belgique	BT2	1214	26-2-2020	25-4-2020
La Belgique	BT3	1234	25-2-2020	25-4-2020
La Belgique	BT4	1250	27-2-2020	27-4-2020
La Belgique	BT5	1072	25-2-2020	25-4-2020
La Belgique	BT6	810	25-2-20	8-4-2020
La Palestine	PT1	1299	5-3-2020	5-5-2020
La Palestine	PT2	1304	5-3-2020	5-5-2020
La Palestine	PT3	1202	1-3-2020	25-4-2020
La Palestine	PT4	1300	3-3-2020	3-5-2020
La Palestine	PT5	1300	3-3-2020	3-5-2020
La Palestine	PT6	1307	29-2-2020	29-4-2020
Ngouleminanga	NT1	1322	8-3-2020	8-5-2020
Ngouleminanga	NT2	1091	8-3-2020	30-4-2020
Ngouleminanga	NT3	1030	9-3-2020	8-5-2020
Ngouleminanga	NT4	1261	9-3-2020	9-5-2020
Ngouleminanga	NT5	654	10-3-2020	8-4-2020
Ngouleminanga	NT6	591	10-3-2020	4-4-2020

To assess the contribution of different animal classes to the soundscape, vocalisation abundance was determined for each vocalising animal. All 1-minute recordings (from all transects) were pulled together. The number of recordings in which an animal class was present was divided by the total number of recordings in order to obtain the vocalisation rate for each class.

To assess how biological sounds vary among sites with differing levels of disturbance, abundance and diversity of vocalisations were compared across study sites. For vocalisation abundance, differences in vocalisation rates per sensor per day across study sites were evaluated for each species. To ensure reliable analysis, only data recorded in all sites on the same day, at the same time, and by sets of sensors with similar spatial designs were used. Sets of sensors were considered to have a similar spatial design in all study sites when the geographical distance between the used sensors was the same (the spatial configuration of the sensors providing data for analysis was consistent across sites). This resulted in data from a total of 30 days. On each of these days, in all sites, one or more sensors with the same spatial design recorded sounds without background noise between 6am–3pm and 9pm–10pm. This totalled 10 min of sound recordings per day. Therefore, calculated vocalisation rates ranged between 0 and 10 for each vocalising species. To compare diversity of vocalising species across sites, sound recordings were only used from times where all acoustic sensors in all three study sites had recorded without background noise. This resulted in a total of 1512 1-minute sound recordings in each site, spread over 23 days. For each vocalising species, the number of sound recordings in which the species was present was determined per site. With these numbers, rarefaction curves were plotted with iNEXT [6]. These curves were extrapolated to larger sample sizes to estimate asymptotic species richness and compare diversity across sites.

To assess the drivers of biological sounds, data on anthropogenic and ecological factors were collected during field surveys. All field surveys were conducted between 8AM and 1PM. In each transect, habitat description and surveys of human activities, mammals (both direct and indirect observations), great ape nests, and birds were conducted. During the surveys, a researcher walked along the transect accompanied by one or more local guides who were able to detect and identify signs. Additionally, data on precipitation, temperature, and humidity were obtained.

For all transects, the vegetation type was described at every marked 50-m interval. For this description, vegetation consisted of 8 different habitat types (mature forest, old secondary forest, young secondary forest, light gap, riparian forest, swamp, old plantation site, and current plantation) based on previous vegetation classifications used in the area [9], [24], [25].

All signs of human activity were recorded within a 2-m range perpendicular to the transect. Both items left by humans, such as cartridges or rubbish, and human constructions, such as trails or traps, were considered human activity. For each observation, the type of human activity, location along the transect (m), and vegetation type were noted. Human activity surveys were conducted twice for every transect, with one month in between surveys. Signs were removed from the transect after counting. Signs that were impossible to remove, such as human trails, were marked during the first survey to avoid recounting during the second survey.

All signs of animal activity present within 2-m on either side of the transect were recorded. The local guide indicated the type of animal sign (e.g., footprint, dung, feeding remain) and the local name of the corresponding animal species. For each observation, the location along the transect (m), perpendicular distance from the transect (m), vegetation type, canopy openness, understorey openness, and horizontal visibility (m) were recorded. Canopy openness (open, average, or closed), understorey openness (open, average, or closed), and horizontal visibility (m) were visually estimated. The openness of the canopy and understorey were always classified as open, average or closed. Indirect mammal surveys were conducted twice for each transect, with one month in between surveys. There was no overlap in observations between the surveys, because rainfall washed away all signs counted during the first survey.

All encountered nests of central chimpanzees (*Pan troglodytes troglodytes*) and western lowland gorillas (*Gorilla gorilla gorilla*) were recorded. The local guide identified the local names of the plants used to construct the nests. Furthermore, the nests were counted and, for each nest, age, location along the transect (m), perpendicular distance from the transect (m), and diameter (cm) were recorded. To reliably distinguish between central chimpanzee and western lowland gorilla nests, several criteria were used. First, fresh nests could be distinguished based on the presence of characteristics like footprints, urine, hairs, and feces [27]. Furthermore, western lowland gorillas commonly sleep in nests on the ground, whereas central chimpanzees tend to build their nests in trees [11], [22]. Consequently, nests lacking clear signs were distinguished based on their height. However, central chimpanzee ground nesting occurs at a low rate in the area [19]. Therefore, nest groups containing at least one nest in a tree at >2-m height were attributed to central chimpanzees, whereas nest groups that were built on the ground or in a tree at <2-m height were attributed to western lowland gorillas [21]. For western lowland gorilla nests, the type of nest was described by the composition of plants used for construction (herbaceous or mix). In turn, for central chimpanzee nests, the type of nest was described by its position in the tree (on the side or on the top). Additionally, the height of the nest was estimated, the height and circumference of the tree were estimated, and the tree was checked for fruits. Finally, vegetation type, canopy openness, understorey openness, horizontal visibility (m), and coordinates were noted for each nest. Great ape surveys were conducted twice for each transect, with one month in between surveys. All nests found during the first survey were marked to avoid overlap with already recorded nests during the second survey.

To avoid disturbing animals before they were observed, observers walked along each transect at a speed of approximately 1 km/h. Any direct observation of mammals along the transect was recorded. The local guide indicated the local name of the species and the number of animals seen. Additionally, the location along the transect (m), the distance between the observer and the animal (m), the angle of the observation, vegetation type, canopy openness, understorey openness, and horizontal visibility (m) were noted.

Birds were surveyed using two methods: point counts in fixed stations and direct observations. At each 500-m interval along the transect, a bird point count station was located. This resulted in three count stations per transect (0 m, 500 m, and 1000 m). At every station, the observers waited two minutes to get acquainted with the environment and to neutralise any possible disturbance caused by the arrival of the observers. Thereafter, an initial observation direction was randomly chosen. After two minutes of observing that direction, the observers rotated 90° in a clockwise direction. This resulted in 8 min of effective observation for each station [23]. Additionally, for every count station, the vegetation type, canopy openness, understorey openness, and horizontal visibility (m) were recorded. The local name and number of individuals of each bird species seen or heard in one direction was noted. If a bird species was seen or heard in multiple observing directions at the same count station, it was only counted once because it could be the same individual. Furthermore, when walking the transect between count stations, direct observations of birds were recorded in the same manner as direct mammal surveys. Finally, since time of day and weather condition can affect bird behaviour, bird surveys were not undertaken on rainy or windy days [10].

Data on rainfall, temperature, and humidity were collected in La Belgique and used to describe all sites. Rainfall was measured in daily precipitation (mm), whereas temperature (°C) and humidity (RH) were noted down hourly.

To assess differences between study sites based on data obtained during field surveys, the encounter rate (observations/km) was used. Thus, for every transect, the mean number of observations for each type of field survey data was calculated. To further investigate the influence of the habitat structure, the total length of swamps and terra firma forests (mature forest, old secondary forest, young secondary forest and light gaps together) in the transects was calculated [24]. Thereafter, the total amount of human, mammal, and bird signs in swamps and terra firma forests was determined. Human activity was calculated overall and broken down into hunting signs and other human signs. The encounter rates of mammal and bird signs were used as index of species abundance. As done in a previous study [8], mammals were grouped in seven defined assemblages of species: elephants, carnivores, even-toed ungulates, pangolins, old world monkeys, great apes, and rodents. Consequently, analyses were performed on mammals as a whole, on the defined taxonomical mammal guilds, and at the species level. Since not all mammals are known to vocalise, another analysis was performed for mammal species that were identified in the acoustic recordings [28]. For elephants, carnivores, even-toed ungulates, pangolins, and rodents, indirect mammal survey data were used. Great ape abundance was estimated by nest counts. To avoid possible bias that may have arisen from grouping individual great ape nests into nest sites, the number of individual nests was used over the number of nest sites. Furthermore, direct observations were used for old world monkey abundance estimates. This approach is consistent with methodologies used in previous studies in the area, enabling the guilds and species to be compared [8], [19], [29]. For birds, the encounter rate was also used as an index of abundance; analyses were performed for all birds together and for each identified bird species separately. To evaluate additional anthropogenic factors that might affect biological sounds, ArcGIS was used to measure the shortest straight-line distance (m) between the sound recorders and the closest village and trail. The distance between the recorder and the closest village served as a proxy for the remoteness of the recording location, whereas distance to the nearest trail was used as a measure of accessibility.

To assess how anthropogenic and ecological factors affect biological sounds, obtained values of mammal abundance, bird abundance, human activity, geographical factors, and climatic measurements were used as predictor variables. Furthermore, two dependent variables were calculated. Since we did not have the same number of recordings for each site, these variables were calculated per sensor per day. The first dependent variable is the proportion of files that contained vocal activity. This proportion, used as a proxy for abundance of vocalisations, was calculated by dividing the number of recordings with vocal activity by the total number of recordings per sensor per day. The second dependent variable is the number of species identified per sensor per day. This variable was used as a proxy for bioacoustic diversity. These dependent variables were calculated for all bird species together and all mammal species together. It is important to note that peak acoustic activity in tropical forests soundscapes occurs during dawn and dusk [5]. Troughout the year, sunrise in the research area always occurs after 5am [26]. Therefore, this study defined a day as a 24-hour time span starting at 6am. This way, every day started with dawn and ended with a full night. All obtained data were organized accordingly. Data obtained during field surveys on transects were attributed to the corresponding sensors. Additionally, the percentage of swamp and terra firma forest was calculated per transect, thus per sensor. Note that only observations of dependent variables for which values for all predictor variables were available were used. This resulted in a total of 847 observations.

### Statistical analysis

Rstudio (version 4.0.2.) was used for all statistical analyses. To determine the structure of biological sounds, normality of all processed data, obtained during the identification of sound recordings, was tested per study site using the Shapiro–Wilk's test. Normally distributed data were tested for homogeneity of variances using a Bartlett test. If data for one of the compared study sites followed a non-normal distribution, a Kruskal–Wallis one-way analysis of variance test was always used because this test does not assume equal variances. As post-hoc analysis, to determine which study sites differ significantly, Dunn's multiple comparison test with Benjamini–Hochberg correction was performed [16]. If data for all study sites were normally distributed and showed equal variances, a one-way ANOVA test was used with Tukey Honest Significant Differences post-hoc analysis. For normally distributed data with unequal variances between the study sites, a Welch ANOVA test with Games-Howell post-hoc analysis was performed.

Sound data in this study follow a hierarchical pattern, where moment of recording is nested in the transects, which are nested in the different study sites. However, preliminary multi-level analyses did not result in a need to treat data from different sites and times differently when assessing the drivers of biological sounds. Therefore, to evaluate the effect of anthropogenic and ecological factors on biological sounds, generalised estimating equations (GEE) were used. GEE are an extension of generalised linear models that allow for the analysis of repeated measurements where observations in separate clusters are independent [12]. To assess multicollinearity among the predictor variables, the variance inflation factor (VIF) of each variable was calculated. Variables with VIF >5 were excluded from the analyses [15]. Additionally, correlation analyses for all pairs of quantitative variables were run. Only temperature and humidity were strongly correlated. Since temperature was measured with more precision, humidity was excluded from the analyses. Poisson models for all dependent variables were used to assess dispersion. Since the number of recordings per sensor per day was not always equal, an offset variable (calculated as log of the total amount of recordings per sensor per day) was added to the models. Models with a dispersion statistic of 0.8 < σ_p_ < 1.2 were considered normally dispersed [17]. Bioacoustic diversity of all wildlife together and birds separately was normally dispersed, whereas total abundance of vocalisations and bird vocalisation abundance was underdispersed. Mammal bioacoustic diversity was also underdispersed and mammal vocalisation abundance was overdispersed. For models that showed under- and overdispersion, binomial GEE analyses were run without an offset variable. However, binomial GEE models require the dependent variable to be a proportion, but mammal bioacoustic diversity was represented as a count value. Therefore, these values were divided by the total number of vocalising mammal species identified throughout the study period to obtain a proportion of bioacoustic diversity. For the GEE analyses, the “exchangeable” correlation structure was used and waves were added to maintain the chronological order of the repeated measurements. The fitted GEE models were compared to similar models to which weights were added to account for the different amounts of recordings that were available per sensor per day. The models were compared using the QIC programme to select the GEE model that best fits the dataset [7]. Models without weights proved to fit the dataset better. Therefore, results from these models were saved.
